# Simulation study on matrix acidification of heterogeneous reservoirs based on seepage-acid etching coupled discrete virtual internal bond

**DOI:** 10.1371/journal.pone.0352115

**Published:** 2026-07-31

**Authors:** Yujie Yan, Yanling Wang, Na An

**Affiliations:** 1 School of Petroleum Engineering, China University of Petroleum (East China), Qingdao, People’s Republic of China; 2 Research Institute of Petroleum Engineering, SINOPEC Northwest Company of China Petroleum and Chemical Corporation, Urumqi, Xinjiang, China; 3 Key Laboratory of Enhanced Oil Recovery in Carbonate Fractured-vuggy Reservoirs, SINOPEC, Urumqi, Xinjiang, China; Northeastern University, CHINA

## Abstract

Matrix acidification is one of the important means for carbonate reservoir modification and permeability increase, and the growth behavior of wormhole determines the effect of matrix acidification. In order to have a deeper understanding of the expansion law of acidified wormhole, this paper carried out numerical simulation of matrix acidification based on the seepage-acid etching coupled discrete virtual internal bond model, and analyzed the influence of matrix acidification on the growth and evolution of acid etched wormhole during matrix acidification in heterogeneous reservoirs. It was found that the enhancement of the heterogeneity of matrix porosity will lead to an increase in wormhole branches and complexity in the acidification process, and the corresponding acid breakthrough time will also become longer. When the anisotropy of the reservoir is strong, there are obvious master wormhole growing perpendicular to the anisotropic direction, and the wormhole morphology is relatively simple. Natural cracks have a strong guiding effect on acid etched wormhole. The results of this paper further reveal the growth law of acid etched wormhole pores, which provides a theoretical understanding for the construction of on-site matrix acidification.

## Introduction

Matrix acidizing is a crucial stimulation technique widely applied in carbonate reservoirs to overcome formation damage and enhance oil and gas recovery [1]. During this process, reactive fluids (e.g., hydrochloric acid) are injected into the porous medium at a pressure below the formation fracture pressure. The acid selectively dissolves the rock minerals along the paths of least fluid resistance, leading to the formation of highly conductive, branching channels known as wormholes [2–11]. The growth behavior and morphological evolution of these wormholes directly dictate the success of the matrix acidizing treatment. Therefore, deeply understanding the mechanism of wormhole propagation under various geological and operational conditions is of great engineering significance.

To investigate wormhole evolution, numerous numerical models have been proposed. Hoefner and Fogler introduced the pore network model, which captures the microstructural properties to some extent but suffers from massive computational costs, making field-scale simulations extremely difficult [12]. Alternatively, Panga et al. developed a two-scale continuum model that effectively simulates acid transport and reaction at the Darcy scale [13]. However, with the rapid development of modern reservoir stimulation technologies, such as cyclic thermal shocks and liquid nitrogen (LN2) cryogenic fracturing, rock masses are subjected to extreme physical and chemical impacts, resulting in the generation of highly complex, multi-branched, and discontinuous fracture networks [14,15]. Traditional continuum models face inherent limitations in capturing such highly discontinuous failure processes and complex topological evolutions, as they often require complex mesh regeneration and heavily rely on predefined macroscopic damage criteria.

To overcome the limitations of existing continuum and network models, this paper introduces a seepage-acid etching coupled Discretized Virtual Internal Bond (DVIB) model to simulate the matrix acidification process [16]. The core innovation and necessity of the DVIB approach lie in its microscopic perspective: it discretizes the rock matrix into a lattice of unit cells connected by virtual micro-bonds, mapping the complex 3D multi-physical field coupling (seepage and chemical dissolution) into 1D bond-based formulations via the Cauchy-Born rule. Unlike continuum models, the macroscopic failure and wormhole propagation in DVIB emerge naturally from the degradation and breakage of these micro-bonds (e.g., chemical dissolution increasing bond porosity and permeability), eliminating the need for empirical macroscopic fracture criteria or dynamic mesh tracking. This provides a unique advantage in simulating the spontaneous branching and complex topology of wormholes in heterogeneous media. Based on this novel framework, this study systematically investigates the growth and evolution of acid-etched wormholes in heterogeneous reservoirs, analyzing the specific influences of acid concentration, injection rate, porosity correlation length (heterogeneity), anisotropic effects, and natural fractures.

## Materials and methods

### Simulation of matrix acidification in heterogeneous reservoirs

The Discretized Virtual Internal Bond (DVIB) method is a microscopic lattice model that discretizes the macroscopic continuum into an assembly of unit cells connected by virtual internal bonds. Unlike traditional continuum models, DVIB equates the macroscopic discontinuous deformation and failure of rocks to the tension, shear, and chemical dissolution of these microscopic bonds. To establish the mathematical framework for the reactive flow within these bonds, several fundamental physical assumptions are made: the acid flow within the bonds follows Darcy’s law, the acid-rock dissolution is characterized by first-order reaction kinetics, and the entire process is assumed to be isothermal. It should be noted that to simplify the computational burden, the current model assumes that the chemical damage (i.e., the increase in bond porosity) linearly degrades its mechanical strength, without considering the complex variation in reaction rates among different heterogeneous minerals. Detailed derivations of the DVIB constitutive equations and their theoretical boundaries can be found in previous classical literature [16].

The model is based on Panga et.al. and Zhu et al. for the theoretical derivation of the seepage-mechanical-chemical three-field coupling discrete virtual internal bond method [17–19], the seepage-acid etching coupling equation for the virtual internal bond is:


$$\begin{array}{c} \frac{\partial \phi }{\partial \text{t}}+{\text{s}}_{\text{b}}\frac{\partial \text{P}}{\partial \text{t}}=\frac{\partial }{\partial \text{x}}(\frac{{\text{k}}_{\text{b}}}{\mu }\cdot \frac{\partial \text{P}}{\partial \text{x}}) \end{array}$$

$$\begin{array}{c} \frac{\partial (\text{C}\phi )}{\partial \text{t}}+\frac{\partial \left(\text{C}{\text{u}}_{\text{x}}\right)}{\partial \text{x}}+{\text{k}}_{\text{c}}{\text{a}}_{\text{v}}\left(\text{C}-{\text{C}}_{\text{s}}\right)-\phi {\text{D}}_{\text{b}}\frac{\partial }{\partial \text{x}}\left(\frac{\partial \text{C}}{\partial \text{x}}\right)=\text{0} \end{array}$$ (1)

Wherein, $u_{x}$ is the acid flow velocity in the bond (m/s); P is the acid pressure (Pa); ϕ is the porosity of the bond; $s_{b}$ is the volumetric water storage coefficient of the bond (Pa^-1^), which relates to the fluid release under unit pressure drop; $k_{b}$ is the permeability of the bond (m^2^); μ is the viscosity coefficient of the acid solution (Pa·s); C is the acid concentration in the fluid (mol/m^3^); $C_{s}$ is the acid concentration at the solid-liquid interface (mol/m^3^); $k_{c}$ is the mass transfer coefficient of the acid (m/s), characterizing the diffusion rate of the acid to the rock surface; $a_{v}$ is the specific surface area of rock pores (m^2^/m^3^); and $D_{b}$ is the equivalent diffusion coefficient (m^2^/s) of the bond.

As the acid fluid flows through the bond, it continuously reacts with the rock matrix, consuming the acid and enlarging the void space of bond. To quantitatively describe this dissolution-induced void enlargement, the porosity evolution equation for the bond is derived based on the acid-rock reaction equilibrium and mass conservation:


$$\begin{array}{c} \frac{\partial \phi }{\partial \text{t}}=\frac{\alpha {\text{k}}_{\text{c}}{\text{k}}_{\text{s}}{\text{a}}_{\text{v}}\text{C}}{\rho_{\text{s}}\left({\text{k}}_{\text{c}}+{\text{k}}_{\text{s}}\right)} \end{array}$$ (2)

Wherein, $k_{s}$ is the first-order reaction rate constant at the acid-rock reaction interface (m/s); α is the dissolving power of the acid (kg/mol), representing the mass of solid dissolved per mole of acid; and $\rho_{s}$ is the rock density (kg/m^3^).

**Fig 1:**
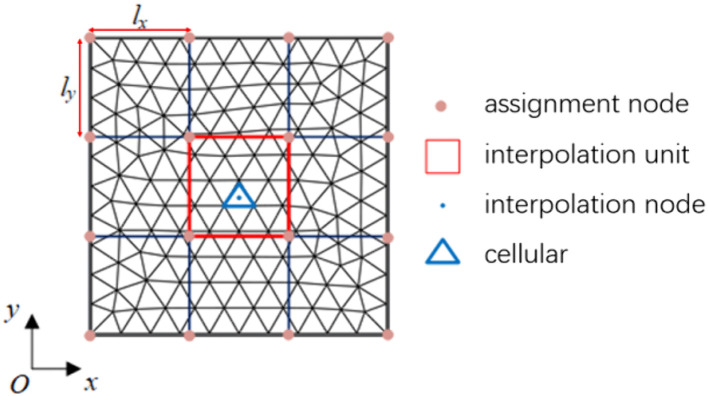
Initial porosity field interpolation method.


The aforementioned mechanisms describe the reactive flow in a single bond. However, actual carbonate reservoirs are rarely homogeneous. To make the simulation reflect real geological conditions, a heterogeneous initial porosity field must be assigned to the computational domain. We utilize an interpolation method based on specific correlation lengths to generate this heterogeneous field (Fig 1). The porosity value of each cell is obtained by interpolating values that obey a certain distribution law assigned to the mesh nodes:

$$\begin{array}{c} \phi (\text{x})=\sum {\text{N}}_{\text{i}}(\text{x})\phi_{\text{i}} \end{array}$$ (3)

Wherein, $N_{i}(x)$ is the interpolation shape function on the node; $\phi_{i}$ is the random initial porosity value assigned to the i-th node; ϕ(x) is the interpolated porosity value of the specific cell; and x indicates the centroid coordinate position of the cell.

Furthermore, sedimentary rocks often exhibit directional heterogeneity due to their natural depositional processes (e.g., bedding planes). To quantitatively describe this directional difference in porosity distribution, the ratio of the correlation lengths in different directions is defined as the anisotropy index χ:


$$\begin{array}{c} \chi =lx/ly \end{array}$$ (4)

Wherein, χ is the anisotropy index of porosity. When χ>1, it indicates that the degree of heterogeneity in the y-direction is greater than that in the x-direction; when χ=1, the formation is isotopically heterogeneous; and when χ<1, the heterogeneity in the y-direction is less than that in the x-direction.

### Model validation

**Fig 2:**
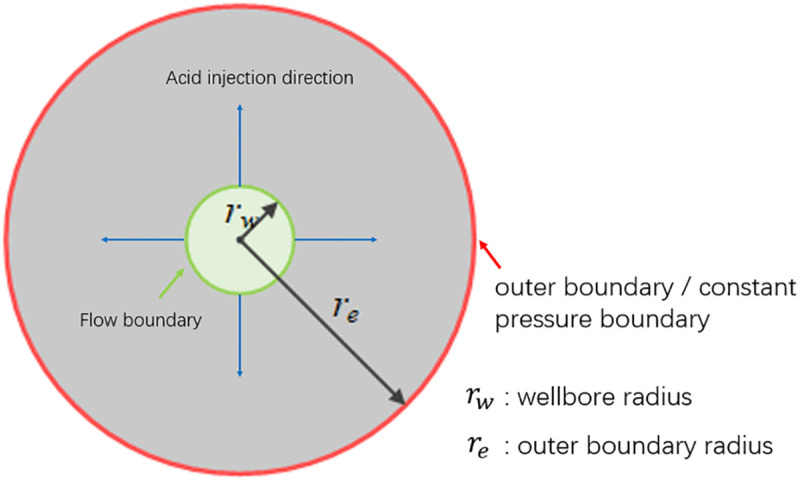
Model settings and boundary conditions.

The model is simulated according to the heterogeneity reservoir, and the wellbore setting is shown in Fig 2. The radial flow model is used to simulate the acid injection process of the wellbore, and the wellbore is set as the flow boundary and concentration boundary, and the outer boundary is the pressure boundary. A triangular grid is used for discretization, the total number of cells is 100336, the total number of nodes is 50496.The acid type is hydrochloric acid and the temperature condition cannot be considered in this model.

To ensure the physical fidelity and numerical accuracy of the simulation, the selection of model parameters and grid resolution was rigorously justified. The physicochemical parameters listed in Table 1, such as the reaction rate constant ($k_{s}$) and diffusion coefficients, were adopted from standard experimental data on carbonate matrix acidizing reported in classic literature [10,11] to represent typical calcite-HCl reaction systems. Furthermore, a grid sensitivity analysis was conducted prior to the production runs. The results indicated that the current unstructured triangular mesh (with a total of 100,336 cells) provides an optimal balance between computational efficiency and accuracy, maintaining the relative error of the acid breakthrough volume below 2% compared to finer mesh resolution.

**Table 1:** The basic parameters used in acidification simulations.

ϕ_0_	Δϕ	k_0_	k_s_	ρ_f_	ρ_s_	s
0.3	0.15	1 × 10^-15^	2 × 10^−3^	1000	2500	0
**α**	**r**_**p0**_	**a**_**v0**_	**μ**	**D**_**mol**_	**l**_**x**_	**l**_**y**_
0.05	1.0 × 10^−6^	5000	0.01	3.0 × 10^−9^	0.01	0.01

Fig 2 shows the computational model and boundary condition settings for the matrix acidizing simulation. A radial flow configuration is adopted, where rw denotes the wellbore radius serving as the flow and concentration boundary, and re denotes the outer boundary radius assigned as a constant-pressure boundary. Acid is injected radially outward from the wellbore, simulating the coupled processes of seepage, diffusion, and acid–rock reaction in a heterogeneous reservoir.

The acid concentration C_0_ = 3000 mol/m^3^, 5000 mol/m^3^ and 8000 mol/m^3^ were taken respectively, and the injection rate was 3.0 × 10^−3^ m^3^/s.

The acid breakthrough volume PVBT can be calculated by the following formula.


$$\begin{array}{c} {PV}_{BT}=V_{inj}/\left(\Phi_{\text{0}}\cdot V\right) \end{array}$$ (5)

**Fig 3:**
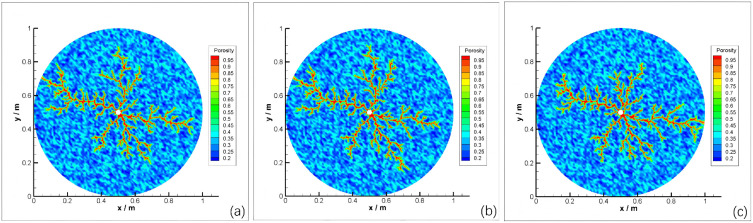
Model validation of porosity clouds at different acid concentrations. (a) C_0_ = 3000 mol/m^3^, t = 200s; (b) C_0_ = 5000 mol/m^3^, t = 125s; (c) C_0_ = 8000 mol/m^3^, t = 75s.

The porosity cloud of the acid breakthrough time at different acid concentrations is shown in Fig 3.

As shown in Fig 3, increasing the acid concentration significantly accelerates wormhole propagation. The color scale represents porosity magnitude: red indicates the highest porosity (fully dissolved regions, approaching 0.95) and blue indicates the lowest (undissolved matrix near the initial value of 0.2). The white circular region at the center represents the wellbore. Quantitatively, when the acid concentration rises from 3000 mol/m^3^ (Fig 3a) to 8000 mol/m^3^ (Fig 3c), the acid breakthrough time decreases drastically from 200 s to 75 s, representing a reduction of 62.5%.

While the porosity clouds in Fig 3 provide a qualitative verification of wormhole patterns, a rigorous quantitative validation is essential to assess the calculation accuracy of the proposed HC-DVIB framework. To this end, we conducted a benchmark comparison against the established two-scale continuum model results reported by Panga et al. under linear flow conditions [11]. The pore volume to breakthrough ($\text{P}{\text{V}}_{\text{BT}}$), which serves as a key quantitative indicator of acidizing efficiency, was plotted against the injection rate to characterize the dissolution regimes.

**Fig 4:**
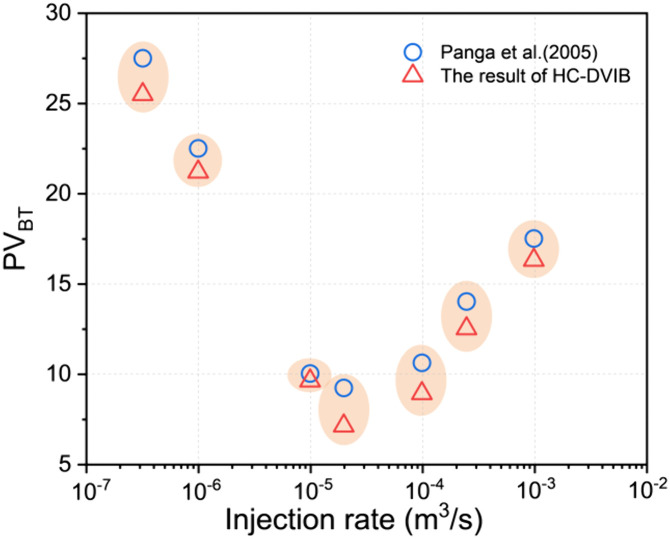
Comparison of the wormhole propagation efficiency curve.

As illustrated in Fig 4, the simulation results of the HC-DVIB model (represented by red triangles) show high consistency with the classic data from Panga et al. (blue circles). Specifically, the model accurately captures the characteristic optimal injection rate — the minimum point on the curve where acid consumption is lowest. The deviation in the critical $\text{P}{\text{V}}_{\text{BT}}$ value is within a reasonable error range, demonstrating that the discrete lattice-based DVIB framework can reproduce the physical behaviors predicted by traditional continuum models with high fidelity.

**Fig 5:**
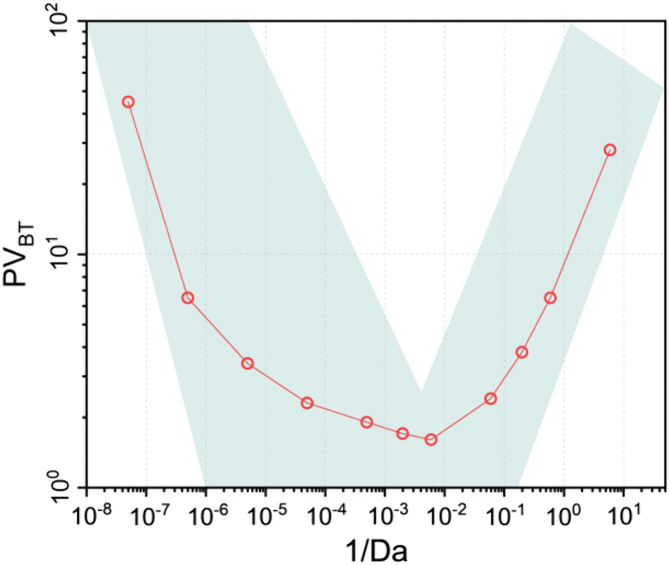
The variation of pore volume to breakthrough with the inverse of the Damköhler number.

Furthermore, to quantify the acidizing efficiency under the radial flow conditions specific to this study, the breakthrough curve was analyzed as shown in Fig 5.

Fig 5 reveals that the wormhole efficiency curve under radial conditions exhibits a typical “V-shape” trend. The curve clearly identifies the optimal operating window (around $\text{1}/\text{Da}\approx {\text{10}}^{-\text{2}}\sim {\text{10}}^{-\text{1}}$) where the dominant worm holing regime occurs. When the injection rate deviates from this optimal value (either too low, leading to face dissolution, or too high, leading to uniform dissolution), the acid consumption ($\text{P}{\text{V}}_{\text{BT}}$) increases significantly. These quantitative curves provide strong justification for the parameter selection in the subsequent sensitivity analyses.

### Heterogeneous reservoir matrix acidification

**Fig 6:**
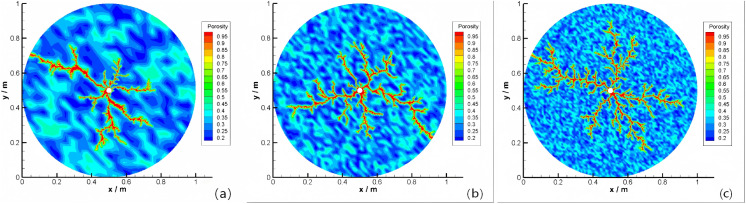
Porosity cloud map of different porosity distributions associated scale. (a) (*l*_x,_ *l*_*y*_) = (0.04, 0.04) m, t = 80s; (b) (*l*_*x,*_ *l*_*y*_) = (0.02, 0.02) m, t = 100s; (c) (*l*_*x,*_ *l*_*y*_) = (0.01, 0.01) m, t = 125s.

In order to reflect the influence of the strength of heterogeneity on the growth process of wormhole under the condition of isotropic equivalence, the morphology of wormhole at the time of acid breakthrough is shown in Fig 6.

Fig 6 illustrates the impact of the porosity correlation length (${\text{l}}_{\text{x}},{\text{l}}_{\text{y}}$) on wormhole propagation. As the correlation length decreases (representing an increase in heterogeneity intensity), the wormhole morphology shifts from a single dominant channel to a highly branched network. Quantitatively, when the correlation length is reduced from 0.04 m (Fig 6a) to 0.01 m (Fig 6c), the acid breakthrough time extends significantly from 80 s to 125 s, representing an increase of 56.3%.

**Fig 7:**
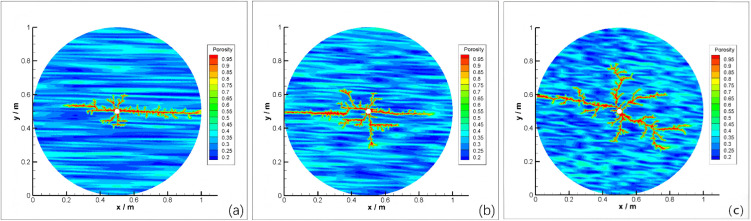
Porosity cloud map of anisotropic effects with different intensities. (a) (*l_x,_* *l_y_*) = (0.20, 0.01) m, t = 45s; (b) (*l_x,_* *l_y_*) = (0.10, 0.01) m, t = 60s; (c) (*l_x,_ l*_*y*_) = (0.05, 0.01) m, t = 90s.

The anisotropy intensity (χ) significantly governs the wormhole propagation efficiency. As indicated in Fig 7, when the reservoir exhibits strong anisotropy (χ=20), a single dominant wormhole rapidly penetrates the formation in just 45 s. In contrast, as the anisotropy weakens to χ=5 (Fig 7c), the lack of strong directional guidance leads to increased ramification, extending the breakthrough time to 90 s—a 100% increase in injection time.

### Heterogeneous reservoir matrix acidification under natural fractures

Natural fractures are also an important cause of reservoir heterogeneity, which has a significant impact on the growth of acid etched wormhole pores. In order to simulate the effect of natural cracks on the formation of matrix acidified wormhole pores, the cracks are directly embedded based on the background grid, and the equivalent permeability of the bond cells cut by the cracks is:


$$\begin{array}{c} k_{f}=\frac{\omega^{\text{3}}}{\text{1}\text{2}c\sqrt{A}} \end{array}$$

$$\begin{array}{c} k_{b}=k_{m}+k_{f} \end{array}$$ (6)

**Fig 8:**
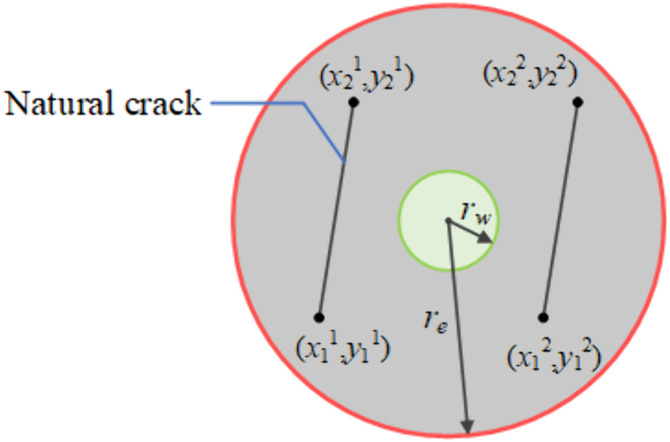
Calculation model with natural cracks.

**Fig 9:**
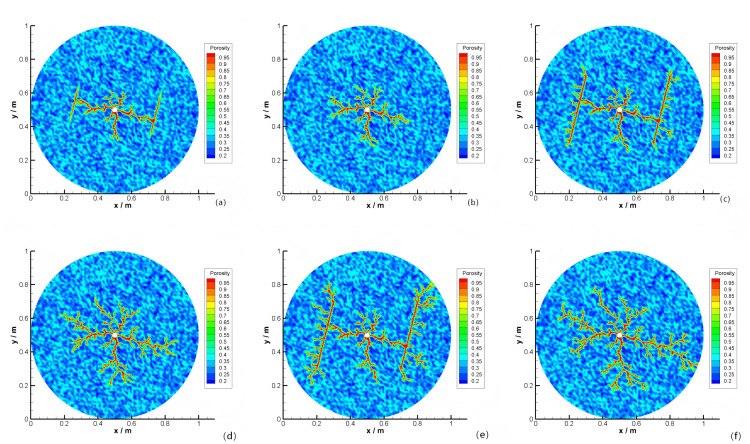
Cloud map of porosity with or without natural cracks. (a) t = 45s, natural cracks; (b) t = 45s, no natural cracks; (c) t = 80s, natural cracks; (d) t = 80s, no natural cracks; (e) t = 110s, natural cracks; (f) t = 110s, no natural cracks.

Among them, kf is the permeability caused by the fracture increases, and the cube law is satisfied; c is the cell shape parameter, for triangular elements c ≈ 1.529; A is the cell volume parameter, which is the triangle element area for this example; km is the permeability of the matrix, w is the crack opening. The calculation model of the preset natural crack is shown in Fig 8, and the coordinates of the two fractures are $\left[x_{\text{1}}^{\text{1}},\;y_{\text{1}}^{\text{1}},\;x_{\text{2}}^{\text{1}},\;y_{\text{2}}^{\text{1}}\right]$ = [0.7,0.3,0.8,0.7] and $\left[x_{\text{1}}^{\text{2}},\;y_{\text{1}}^{\text{2}},\;x_{\text{2}}^{\text{2}},\;y_{\text{2}}^{\text{2}}\right]$ = [0.2,0.3,0.3,0.7], where the coordinates of the natural crack start node and the node coordinate of the end point of the natural crack are respectively $\left(x_{\text{1}}^{\text{i}},\;y_{\text{1}}^{\text{i}}\right)$ and $\left(x_{\text{2}}^{\text{i}},\;y_{\text{2}}^{\text{i}}\right)$. The initial open*^i^*ng of the natural crack is 0.005m. The simulation results of the model with or without natural cracks are shown in Fig 9.

Fig 8 shows the computational model with two pre-embedded natural fractures superimposed on the background radial-flow mesh. The endpoint coordinates of the two fractures are [0.7, 0.3, 0.8, 0.7] and [0.2, 0.3, 0.3, 0.7], respectively, with an initial aperture of 0.005 m for both. The permeability of cells intersected by fractures is treated by an equivalent approach, in which the fracture-induced permeability contribution (following the cubic law) is superimposed onto the matrix permeability, enabling accurate representation of fracture flow characteristics without remeshing.

Fig 9 presents a comparative visualization of porosity distributions at three time steps (t = 45 s, 80 s, and 110 s) for cases with natural fractures and without. In the fractured case, wormholes initially propagate along high-permeability preferential paths; upon encountering a natural fracture, they are captured and redirected along the fracture until it is fully dissolved. During this dissolution process, numerous small wormholes nucleate around the fracture, connecting a broader extent of the rock matrix. In the unfractured case, wormhole paths are governed solely by the heterogeneous porosity field and are more dispersed. The comparison clearly reveals the strong capturing and guiding mechanism exerted by natural fractures on acid-etched wormhole propagation.

### Field application significance

The numerical model of acid-etched wormhole propagation developed in this study provides significant guidance for field-scale acidizing design and parameter optimization, and its key predictions have been preliminarily validated by field monitoring results.

**Fig 10:**
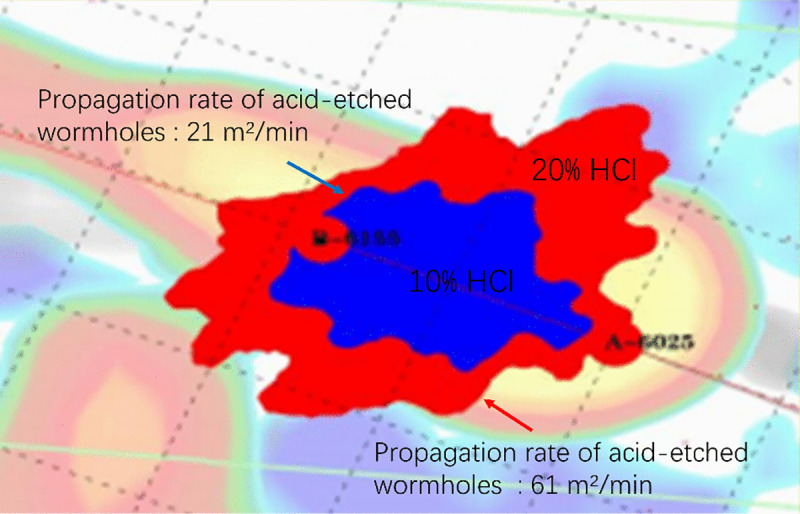
Monitoring the rate of wormhole formation at different acid injection concentrations using magnetotelluric method.

Numerical simulations demonstrate that acid concentration exerts a pronounced influence on wormhole propagation rate: high-concentration acid (20% HCl) yields a substantially higher wormhole propagation rate (61 m²/min) compared to low-concentration acid (10% HCl, 21 m²/min). This finding carries direct practical value for field parameter design. When the stimulation objective requires a large swept volume, higher acid concentrations should be preferred to maximize propagation efficiency. Conversely, in formations with high acid sensitivity or where precise control of the stimulation extent is required, reducing acid concentration can mitigate the risk of excessive dissolution. These simulation predictions are qualitatively corroborated by wide-area magnetotelluric (MT) field monitoring (Fig 10), where the spatial distribution of wormholes under different acid concentrations is consistent with model outputs, thereby providing a reliable basis for acid system selection in field operations.

Fig 10 presents field-scale wormhole formation rate distributions obtained by wide-area magnetotelluric monitoring under two acid injection concentrations. The blue area corresponds to a low-concentration treatment (10 wt%), with a wormhole formation rate of approximately 21 m²/min; the red area corresponds to a high-concentration treatment (20 wt%), yielding a rate of approximately 61 m²/min.

**Fig 11:**
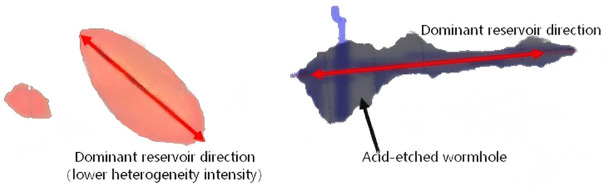
Monitoring the bidirectional, non equivalent, and heterogeneous wormhole generation rate using magnetotelluric method.

Numerical simulations reveal that along the dominant reservoir direction governed by depositional processes—characterized by a larger porosity correlation length and relatively weaker heterogeneity—acid-etched wormholes propagate significantly farther, whereas propagation is suppressed in directions of stronger heterogeneity. This anisotropic behavior has important implications for the directional design of acidizing treatments. During well placement and perforation orientation selection, the spatial distribution of depositional architecture and porosity heterogeneity should be carefully incorporated, with the primary stimulation direction aligned to the dominant reservoir flow direction to maximize the acid-stimulated volume and improve treatment efficiency. Field MT monitoring results (Fig 11) show that the morphology of acid-etched wormholes is in good agreement with the dominant reservoir direction, validating the model’s predictive capability for anisotropic wormhole propagation and providing a reference for acidizing design in analogous reservoirs.

Fig 11 presents field magnetotelluric monitoring results showing wormhole propagation morphology under anisotropic reservoir conditions. The elliptical outline in the left panel represents the dominant porosity orientation formed by sedimentary processes, indicating the direction of weaker heterogeneity and lower seepage resistance. The right panel shows that a prominent dominant wormhole extends up to 50 m along the preferential direction, while propagation perpendicular to this direction is severely limited to approximately 10 m.

**Fig 12:**
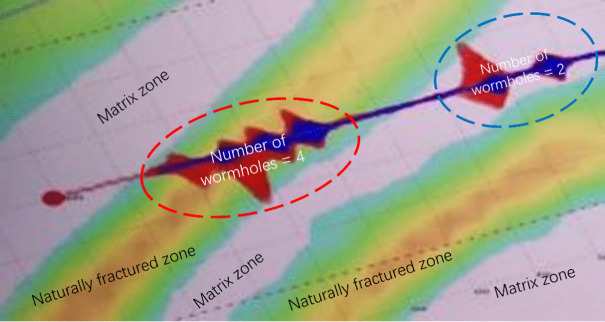
The influence of magnetotelluric monitoring of natural cracks on the rate of wormhole formation.

Simulation results further indicate that natural fractures exert a pronounced directional “capturing” effect on wormhole development—wormholes preferentially propagate along natural fracture orientations, with greater numbers and larger extents in fractured zones compared to matrix-dominated regions. Understanding this mechanism is of direct relevance to field treatment decisions. On one hand, injecting acid in naturally fractured intervals can leverage the enhanced conductivity of fractures to amplify the stimulation effect. On the other hand, caution is warranted against premature acid breakthrough along fractures, which may confine the effective stimulation volume to the fracture vicinity and compromise overall treatment uniformity. Field MT monitoring results (Fig 12) show that the number of wormholes in the naturally fractured zone (yellow-green background) reaches 4, while only 2 small wormholes are observed in the rock matrix zone (white background). This observation is consistent with the model’s prediction of fracture-controlled preferential wormhole development, further supporting the field applicability of the proposed model.

Fig 12 presents field magnetotelluric monitoring results illustrating the influence of natural fractures on wormhole formation. The yellow-green background region represents a zone of well-developed natural fractures, where up to 4 wormholes are observed propagating along the fracture orientation. The white background represents the intact matrix zone without natural fractures, where only 2 smaller wormholes are formed under equivalent treatment conditions. The contrast demonstrates that natural fractures exert a strong guiding and promoting effect on acid-etched wormhole development, making fracture characterization a critical factor in acidizing program design.

## Discussion

To fundamentally understand the quantitative relationship between the acid diversion mechanism and the evolution of reaction surface area during multi-branch wormhole formation, it is necessary to analyze the process from a fluid mechanics perspective, specifically through the lens of the Dam Köhler number (Da). The Da number characterizes the competition between the acid-rock chemical reaction rate and the convective mass transport rate. When the acid concentration is high (or the injection rate is extremely low), the system shifts towards a reaction-limited regime characterized by a large Da. In this state, the acid is rapidly consumed at the pore fluid-solid interfaces immediately upon contact, failing to penetrate deeper into the matrix via convection. This intense local reaction restricts the ‘acid diversion’ effect, leading to a smaller overall reaction surface area and the formation of a few, thick, but poorly penetrating channels. Conversely, under optimal Da conditions, the convective transport outpaces the local reaction rate, allowing live acid to be effectively diverted into multiple high-permeability microscopic pathways. This fluid diversion mechanism significantly increases the effective reaction surface area, fostering the development of a complex, multi-branched wormhole network.

Furthermore, the inherent geological heterogeneity and anisotropy strongly dictate the spatial distribution of these flow channels. As observed in our simulations, a decrease in the porosity correlation length intensifies the local permeability contrast. This heterogeneity disrupts the uniform advancement of the fluid front, triggering severe acid diversion into numerous micro-pores. Consequently, the acid volume is continuously consumed to create new branches (increasing the morphological complexity and reaction surface area) rather than extending the master wormhole, which thoroughly explains the significant extension in breakthrough time. On the other hand, the anisotropic effects provide macroscopic flow guidance. Under strong vertical anisotropy (χ=20), the permeability contrast heavily favors horizontal advection, forcing the fluid to flow predominantly along the bedding direction. This restricts transverse acid diversion, minimizing side-branching and leading to rapid, straight penetration. As the formation approaches isotropic conditions (χ=5), the lack of directional guidance allows the fluid pressure to distribute more uniformly, increasing the radial acid invasion and resulting in a highly tortuous, multi-directional wormhole network.

## Limitations and future works

While the proposed HC-DVIB model effectively captures the wormhole propagation behaviors in heterogeneous reservoirs, several limitations should be noted. First, the current framework operates under isothermal conditions, neglecting the potential impact of geothermal gradients on acid reaction kinetics and fluid viscosity. Although the injected fluid often reaches local thermal equilibrium rapidly, temperature sensitivity could be significant in deep, high-temperature reservoirs. Second, the chemical damage is simplified as a porosity evolution process without coupling the dynamic fracture closure induced by effective stress, which may influence conductivity under high closure stress conditions. Third, the rock matrix is treated as a single-mineral system, whereas actual carbonates may exhibit differential reaction rates due to mineralogical heterogeneity. Future work will aim to extend the DVIB framework to a fully coupled Thermal-Hydraulic-Mechanical-Chemical (THMC) model to address these multiphysics interactions comprehensively.

## Conclusions

Combined with the seepage-acid etching DVIB model, this paper focuses on the influence of the growth and evolution of acid etching wormhole pores during the acidification of heterogeneous reservoir matrix, and the following conclusions can be drawn:

(1) The porosity correlation length decreased, that is, the isotropically equivalent heterogeneity increased, the branches of wormhole increased, the degree of morphological complexity increased, and the acid breakthrough time became longer;
(2) The reservoir has strong anisotropy, and there are obvious master wormhole growing perpendicular to the anisotropic direction, and the wormhole morphology is relatively simple, while when the anisotropy is weak, there are less obvious master control wormhole growing perpendicular to the anisotropic direction, and the wormhole morphology is more complex;
(3) The crack has a strong guiding effect on the acid etched wormhole, and when the wormhole is captured by the natural crack, it will grow along the natural crack until the natural crack is completely dissolved.

The results of this paper further reveal the growth law of acid etched wormhole pores, which provides a theoretical understanding for the construction of on-site matrix acidification.The limitation of this study is that the temperature field cannot be considered, and the crack seepage model is relatively simple. Further improvements are needed in the future to make the model more widely applicable.

## Supporting information

S1 File Data underlying Fig 3. This Excel file contains the data used to generate Fig 3. (XLSX)

S2 File Data underlying Fig 4. This Excel file contains the data used to generate Fig 4. (XLSX)

S3 File Data underlying Fig 5. This Excel file contains the data used to generate Fig 5. (XLSX)

S4 File Data underlying Fig 6. This Excel file contains the data used to generate Fig 6. (XLSX)

S5 File Data underlying Fig 7. This Excel file contains the data used to generate Fig 7. (XLSX)

S6 File Data underlying Fig 9. This Excel file contains the data used to generate Fig 9. (XLSX)

S7 File Supporting code. This ZIP file contains all code files used in this study.
(ZIP)
